# Safety and feasibility of high dose stress dobutamine mri very early after acute myocardial infarction

**DOI:** 10.1186/1532-429X-13-S1-P88

**Published:** 2011-02-02

**Authors:** Michael Mamone, Stephanie Lehrke, Dirk Lossnitzer, Grigorios Korosoglou, Evangelos Giannitsis, Hugo A Katus, Henning Steen

**Affiliations:** 1 University Heidelberg, Heidelberg, Germany

## Introduction

Dobutamine stress studies after acute ST or Non-ST elevation myocardial infarction (STEMI, NSTEMI, AMI) have prognostic implications for future cardiovascular events and therefore have an impact on the indicated therapy by identifying high-risk patients. Dobutamine side effects early after AMI have been extensively reported. Namely malignant arrhythmias are feared, since mostly the initiated beta blocker therapy was interrupted for stress studies to achieve the required sub-maximal heart rate increase.

## Purpose

To date, no data exists on the feasibility of dobutamine stress MRI (DSMR) in patients (pts.) very early after AMI with continued beta-blocker therapy. Therefore we sought to investigate the side effects and safety of DSMRI early after AMI.

## Material and methods

144 pts. with a first uncomplicated AMI underwent DSMR (max. 40mg/kg bw/min plus atropine if needed) for detection of ischemia under continuous high dose beta blocker therapy. Studies were terminated after reaching sub-maximal heart rate (calculated by 200-age-10% beats-per-minute(bpm)) or typical chest-pain/dyspnoea/malignant arrhythmia. Stress-induced wall motion deterioration was considered as positive DSMR. DSMR was conducted in a 1.5T whole body MRI (Philips), vital parameters like heart rate, blood pressure and -saturation were monitored continuously.

## Results

We studied 63 STEMI and 81 NSTEMI (mean age 66±12, 15% female). 36pts. had a one-, 26pts. a two- and 79 pts. three-vessel disease with 127 stents implanted. Maximal Troponin T was 2.96±3.68ng/dl. NT-pro BNP was 2422±3838ng/l. Clinical reasons for DSMR were assessment of a) ischemia (126 pts.) and b) viability (18 pts). DSMR was conducted in 94% successfully. Sub-maximal heart rate was reached in approximately 85% of pts (from 64±12to115±21/bpm). 81% of pts. received the maximal 40-dobutamine dose pre-discharge (4.9±2.1days after AMI). 40% obtained additional atropine (max. 2mg) regardless whether STEMI or NSTEMI had occurred. During DSMR 18 pts. reported chest pain, 8 pts had dyspnoea. In 16 pts. DSMR detected new wall motion abnormalities suspicious of myocardial ischemia.

## Conclusions

After AMI conduction of DSMR was safe and well tolerated by all pts. Potentially due to the continued beta-blocker therapy, no malignant arrhythmias occurred although 85% of pts. reached the required sub-maximal heart rate. Since stress studies after AMI have prognostic implications for future cardiovascular events and are therefore clinically necessary, high dose DSMR can be conducted safely under beta-blocker therapy.

